# Hearing consequences in Gjb2 knock-in mice: implications for human p.V37I mutation

**DOI:** 10.18632/aging.102246

**Published:** 2019-09-27

**Authors:** Xin Lin, Gen Li, Yu Zhang, Jingjing Zhao, Jiawen Lu, Yunge Gao, Huihui Liu, Geng-Lin Li, Tao Yang, Lei Song, Hao Wu

**Affiliations:** 1 Department of Otolaryngology-Head and Neck Surgery, Shanghai Ninth People’s Hospital, Shanghai Jiao Tong University School of Medicine, Shanghai 200011, China; 2 Ear Institute, Shanghai Jiao Tong University School of Medicine, Shanghai 200125, China; 3 Shanghai Key Laboratory of Translational Medicine on Ear and Nose diseases, Shanghai 200125, China

**Keywords:** GJB2, age-related hearing loss, potassium recycling, environmental stress, hair cells

## Abstract

Human p.V37I mutation of *GJB2* gene was strongly correlated with late-onset progressive hearing loss, especially among East Asia populations. We generated a knock-in mouse model based on human p.V37I variant (c.109G>A) that recapitulated the human phenotype. Cochlear pathology revealed no significant hair cell loss, stria vascularis atrophy or spiral ganglion neuron loss, but a significant change in the length of gap junction plaques, which may have contributed to the observed mild endocochlear potential (EP) drop in homozygous mice lasting lifetime. The cochlear amplification in homozygous mice was compromised, but outer hair cells’ function remained unchanged, indicating that the reduced amplification was EP- rather than prestin-generated. In addition to ABR threshold elevation, ABR wave I latencies were also prolonged in aged homozygous animals. We found in homozygous IHCs a significant increase in I_Ca_ but no change in Ca^2+^ efficiency in triggering exocytosis. Environmental insults such as noise exposure, middle ear injection of KCl solution and systemic application of furosemide all exacerbated the pathological phenotype in homozygous mice. We conclude that this *Gjb2* mutation-induced hearing loss results from 1) reduced cochlear amplifier caused by lowered EP, 2) IHCs excitotoxicity associated with potassium accumulation around hair cells, and 3) progression induced by environmental insults.

## INTRODUCTION

Congenital hearing loss is the most common hereditary disease in human with a morbidity rate of 2-3‰ [1–3]. Mutations in *GJB2*, which encodes connexin26 (Cx26) protein responsible for building up the gap junction (GJ) with connexin30 (Cx30) to form heterotypic GJ channels in the nonsensory epithelium in the cochlea, are among the most common disease causes [4–9]. *GJB2* is expressed in inner ear supporting cells, stria vascularis, and spiral ligament; it is involved in a series of physiological processes of hearing including cochlear development, the generation of endocochlear potential (EP), active cochlear amplification, second messenger transduction and potassium recycling [10–16]. Our previous studies found that p.V37I mutation of the *GJB2* gene is related to a broad spectrum of hearing phenotypes in human. Approximately 65% of the patients with *GJB2* p.V37I mutation had congenital hearing loss and the remaining 35% had a delayed disease onset. The degree of hearing loss also varied in patients, ranging from normal hearing to profound hearing loss, with the severity of hearing loss strongly correlated with age [17, 18].

Due to the high prevalence of *GJB2* mutation in East Asia population with nearly all ethnic background, it is becoming a primary focus of genetic screening [19–21]. However, there is a lack of understanding of the disease prognosis, and prevention is currently unavailable. As the disease progression varies in time of onset and the severity of the hearing loss, we hypothesize that *GJB2* mutations alter inner ear’s susceptibility to environmental stresses, the exposure to which accumulates over time. Since a large number of *GJB2* mutation patients have late onset of hearing loss, pinpointing the molecular mechanism of the mutation could help formulate strategies to prevent or delay hearing loss in this specific patient population [22] and develop potential treatments.

*In vitro* studies found that p.V37I mutation only partially reduced the permeability of GJs and dysfunctional hemichannels [23, 24]. In other forms of *Gjb2* mutations, EP changes were found to be associated with hearing losses [25]. However, whether p.V37I mutation changes EP and whether/how it consequently affects hearing *in vivo* is unclear. To further investigate the underlying disease mechanism of p.V37I mutation, we previously generated a knock-in (KI) mouse model with the same single base-pair change found in human p.V37I variant [26]. This single base pair KI mouse model could serve as a platform to explore future genetic rescue such as base editing, a safer gene editing method [27, 28]; and to evaluate the rescue outcome by comparing with chemical interventions in the same animal model.

The knock-in mouse exhibited disease progression pattern analogous to that in patients with p.V37I mutation, with late onset of progressive hearing loss observed. In addition to general hearing loss progression, we also examined the underlying tissue-specific mechanism of the hearing loss. We discovered three pathological changes in the inner ear: a minor change in Cx26 hemichannel morphology, reduced EP, and altered pre-synaptic functions of the inner hair cells (IHCs). Although cochlear function was maintained at normal level in the early life of the mutant animal, this ‘fragile normal’ state was vulnerable to environmental insults. We investigated the impact of three environmental risk factors, noise, ototoxic drug, and the disruption of ionic homeostasis in the cochlea, and found that they all accelerated the disease progression. These identified environmental factors offer insights into potential disease prevention strategies and may guide the future development of therapeutics.

## RESULTS

### General ABR findings: KI mice exhibited late-onset progressive hearing loss with prolonged Wave I latency

**Figure 1:**
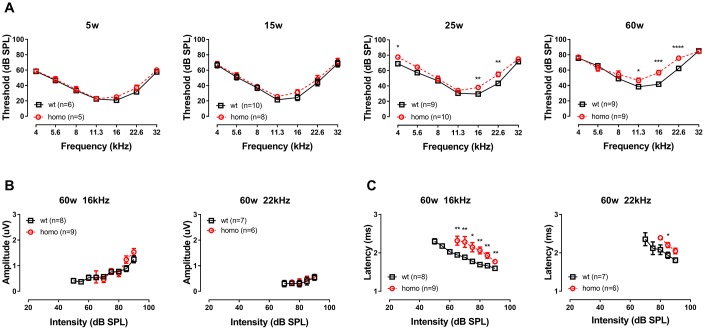
**Auditory Brainstem Response (ABR) threshold and Wave I analysis in KI mice.** (**A**) Auditory Brainstem Responses (ABR) of p.V37I knock-in mice (Homozygous, red dotted line, Mean ± SEM) and age-matched wild-type mice (wt, black line, Mean ± SEM). Significant ABR threshold elevation was not observed until 25 weeks old, which started from 16 kHz (**P=0.005, F_(1.8)_=14.629 at 25 weeks and *** P=0.000137, F_(1,8)_=46.286 at 60 weeks, two-way ANOVA followed by Bonferroni post-test) and 22 kHz (**P=0.002, F_(1,8)_=19.412844 at 25 weeks and **** P=0.000044, F_(1,8)_=64.000 at 60 weeks, two-way ANOVA followed by Bonferroni post-test) gradually expanded to 11 kHz (*P=0.028, F_(1,8)_=7.143 at 60 weeks, two-way ANOVA followed by Bonferroni post-test). A significant ABR threshold elevation in 4 kHz was observed at 25 weeks old (*P=0.010333, F_(1,8)_=11.111, two-way ANOVA followed by Bonferroni post-test) but disappeared at 60 weeks old. ABR Wave I amplitude (**B**) and latency (**C**) of 16kHz and 22.6kHz in 60 week-old homozygous (red dotted line, Mean ± SEM) and wild-type (black line, Mean ± SEM) mice plotted as a function of sound levels. Amplitude did not differ between genotypes (both P>0.05, two-way ANOVA followed by Bonferroni post-test), while latency showed significant prolongation in homozygous mice (*P<0.01, **P<0.001, two-way ANOVA followed by Bonferroni post-test). Animals lacked visible Wave I were excluded for amplitude and latency analysis.

The general hearing threshold measurements revealed a mild form of progressive hearing loss in KI mice starting around 6 months of age. Timeline of disease progression was postponed compared to our previous report on the same animal model [26]. This change may due to the reduced ambient acoustic noise that had resulted from relocated and improved animal housing (no significant ambient noise at 2-100 kHz). In addition to the parallel threshold elevation due to age (monitored up to 60 weeks), homozygous mice also exhibited additional 10-15 dB SPL threshold elevation around mid-high frequency region when compared with their age-matched wild-type controls (Figure 1A, P<0.05 at 11.3, 16 and 22.6 kHz at 60 weeks old, two-way ANOVA followed by Bonferroni post-test). We chose the latest time point of our measurement (60 weeks) at frequencies with significant threshold elevations (16 kHz and 22 kHz) to analyze ABR Wave I and found no difference in Wave I amplitude between the two genotype groups (Figure 1B, both P>0.05, two-way ANOVA followed by Bonferroni post-test), but significantly prolonged latencies in homozygous mice (Figure 1C, **P<0.01 or ***P<0.001, two-way ANOVA followed by Bonferroni post-test). Since ABR wave I amplitude and latency reflect the number of activated, synchronized firing neurons and the timing of synaptic transmission and nerve conduction [29], we directed our focus on two functional aspects: 1) factors that could affect cochlear amplifier, including EP, which drives the ion flow through transduction channels in inner and outer hair cells, and 2) the synaptic function of IHCs, where gating and synaptic release were investigated.

### Tissue-specific pathology revealed mild cochlear morphological and functional changes:

### Cx26 hemichannels remained intact with minor morphological changes

**Figure 2:**
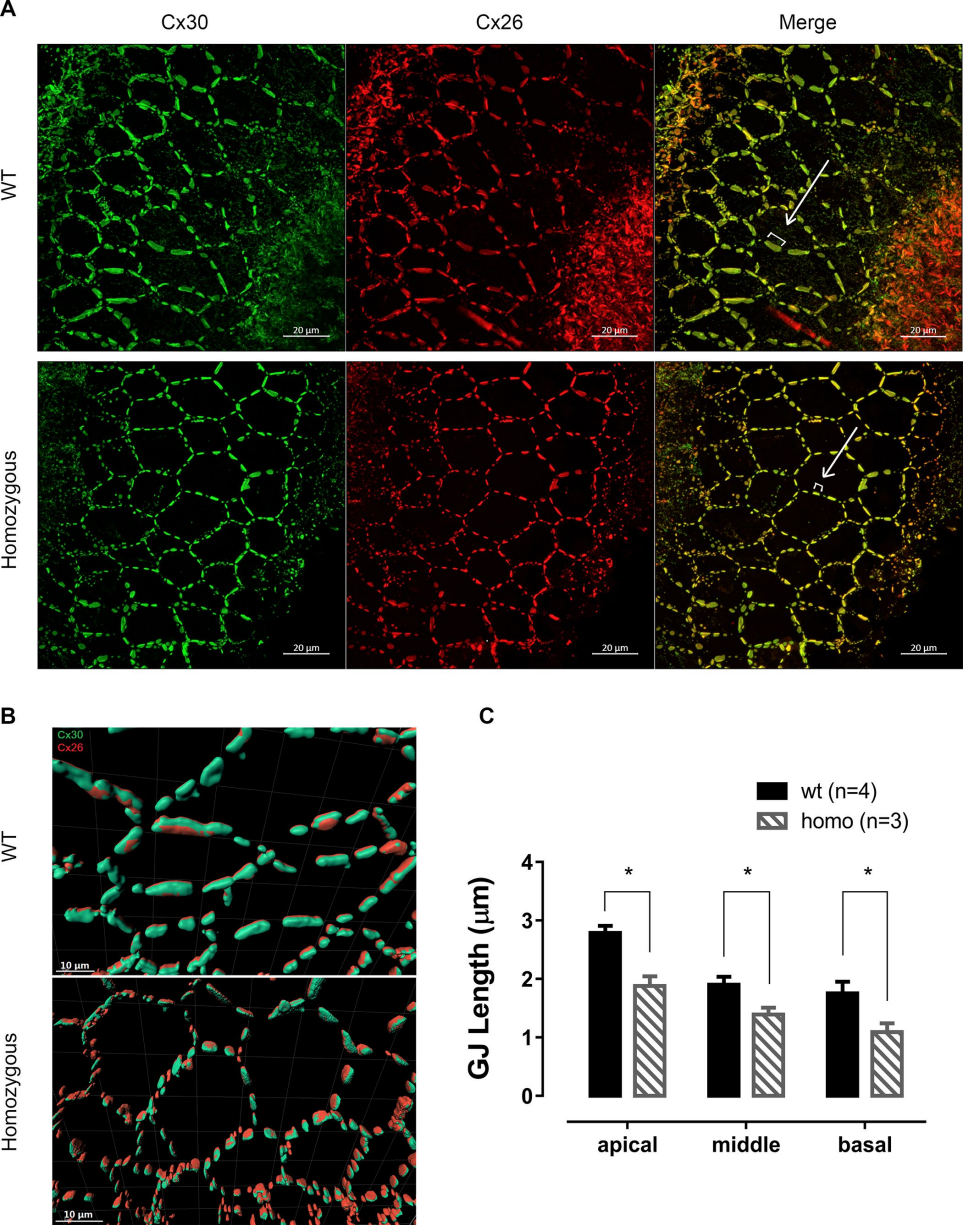
**Connexin expression patterns in senior KI mice.** (**A**) Representative confocal images of connexin 26 (Cx26, red), connexin 30 (Cx30, green) and merged images at the age of 60 weeks from apical turns of the basilar membrane. Cx26 and Cx30 were well expressed on the supporting cell membrane and mostly overlapped, forming a honeycomb-like labeling pattern. White brackets indicate the length of representative Cx plaques measured. Scale Bar, 20 μm. (**B**) Graphs of Imaris 3D-reconstructed images plotted at higher magnification. Cx26 (red) were accurately co-localized with Cx30 (green) in an adjacent parallel pattern. Cx plaques appeared shorter in homozygous supporting cells (Scale Bar, 10 μm). (**C**) Histograms presenting the average length of the CJ plaques, measured as shown in panel A, of both homozygous and wild-type mice (Mean + SEM). GJs were significantly shorter in homozygous mice in every turn (*P<0.05, two-way ANOVA followed by Bonferroni post-test).

Cx26 and Cx30 are the most predominant form of GJs in the cochlea [30, 31]. The immunofluorescent staining of 60 week-old homozygous mice revealed honeycomb-like structures of GJs on the membrane of supporting cells (Figure 2A), where Cx26 precisely co-localized with Cx30 (Figure 2B). By measuring GJ plaques as indicated in Figure 2A, shorter averaged length of GJ plaques was found in homozygous mice (Figure 2C, P<0.05 in all the three turns, two-way ANOVA followed by Bonferroni post-test). This morphological change could have functional consequences such as reduced pore size, causing the reduction of the GJ permeability.

### EP was reduced but remained unchanged throughout the lifespan of KI mice

**Figure 3:**
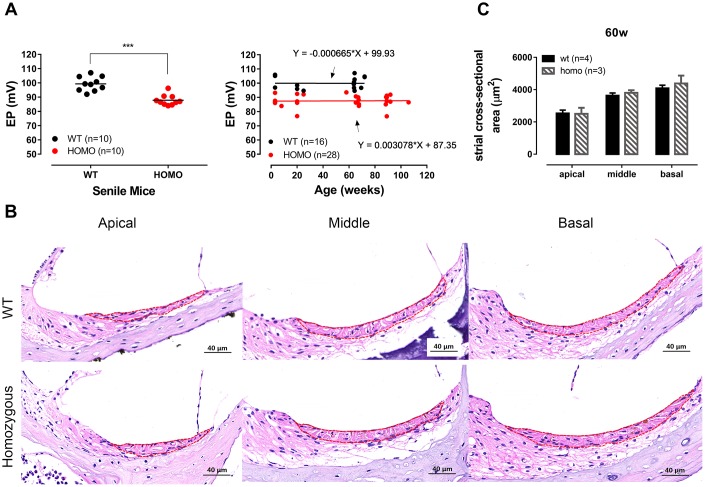
**Endocochlear potential (EP) measurement and Stria Vascularis morphology in KI mice.** (**A**) Significantly reduced EP in senile homozygous mice (ranging from 60 weeks old to 90 weeks old) (Left panel, ***P <0.0001, t=5.876, df=16.6, Student’s unpaired t-test with Welch’s correction). Symbols represent individual measurement and lines are the means. EP reduction of homozygous mice remained stable within the expanded timeline from 3 postnatal weeks to ~2 years (Right panel). Linear regressions showed that slopes of both groups were virtually zero (fitted functions are shown in the right panel, both P>0.05, linear regression). (**B**) Representative H&E staining of Stria vascularis for all three turns with cross-sectional areas outlined for quantitative analysis (scale bar, 40 μm). (**C**) Histogram illustrating the averaged cross-sectional area of Stria Vascularis of 60 weeks old cochleae (Mean + SEM). The cross-sectional area showed no significant difference between wild-type and homozygous mice (P>0.05, two-way ANOVA followed by Bonferroni post-test).

In some *Gjb2* animal models, the characteristic phenotype was the hearing threshold elevation accompanied with EP drop [13, 25, 32–34]. Since GJs are believed to serve as ion diffusion shortcut among supporting cells [10, 35], we reasoned that the subtle morphological difference implied consequences in cochlear homeostasis that could eventually affect the generation of EP, as seen in Cx26 and Cx30 double mutation [13]. When we measured EP at the age of 60 weeks, homozygous mice exhibited a small (~12 mV) but significant reduction (Figure 3A, left panel, P <0.0001, t=5.876, df=16.6, Student’s unpaired t-test with Welch’s correction). To validate this observation, we extended our EP recordings to cover a wider age range and found that the difference of EP was consistent throughout the lifespan. The EP reduction was observed from the end of the third postnatal weeks when both EP and cochlear development just completed [36, 37], and the reduction persisted up to 100 weeks of age (Figure 3A, right panel, both P>0.05, linear regression). Cross-sectional H&E stainings showed no apparent morphology change in Stria Vascularis (SV) in homozygous when compared to the wild-type controls (Figure 3B, 3C, P>0.05, two-way ANOVA followed by Bonferroni post-test), ruling out SV atrophy as the source of EP reduction.

### OHC functions were unchanged but may have operated at an abnormal condition indirectly responsible for the progressive hearing loss of KI mice

Although the slightly reduced EP remained stable throughout the homozygous animals’ lifespan, the reduced driving force through transduction channel could potentially impact the hair cells’ survival and performance, particularly in OHCs [38–40]. To examine whether the observed threshold elevation was the result of the compromised cochlear amplification process, we measured OHC function in the homozygous animals.

**Figure 4:**
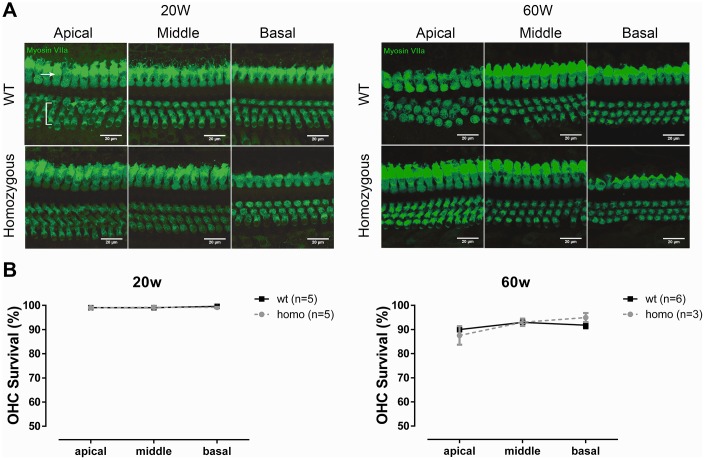
**OHC count in KI mice.** (**A**) Representative confocal images of OHCs and IHCs at the age of 20 weeks and 60 weeks. Bracket indicates three rows of OHCs, and arrowhead points to one row of IHCs (Scale Bar, 20 μm). (**B**) Line graphs illustrating the percentage of OHC survival in every cochlear turn of each group and at each time point. There were fewer losses of OHCs in both homozygous and wild-type mice at 20 weeks old (left panel, P>0.05, two-way ANOVA followed by Bonferroni post-test). Both groups had minor OHC loss at the age of 60 weeks, but no difference between the two groups was found (right panel, P>0.05, two-way ANOVA followed by Bonferroni post-test).

We first evaluated the cochlear morphology to quantify OHC loss. The sensory epitheliums of all three turns were collected at both 20 and 60 weeks for confocal imaging. Increased OHC loss was detected in the older age groups, but the extent of OHC losses were comparable between the two genotype groups at all three turns measured (Figure 4A, 4B, all P>0.05, two-way ANOVA followed by Bonferroni post-test), thus ruling out the loss of OHCs as the source of observed hearing phenotype.

**Figure 5:**
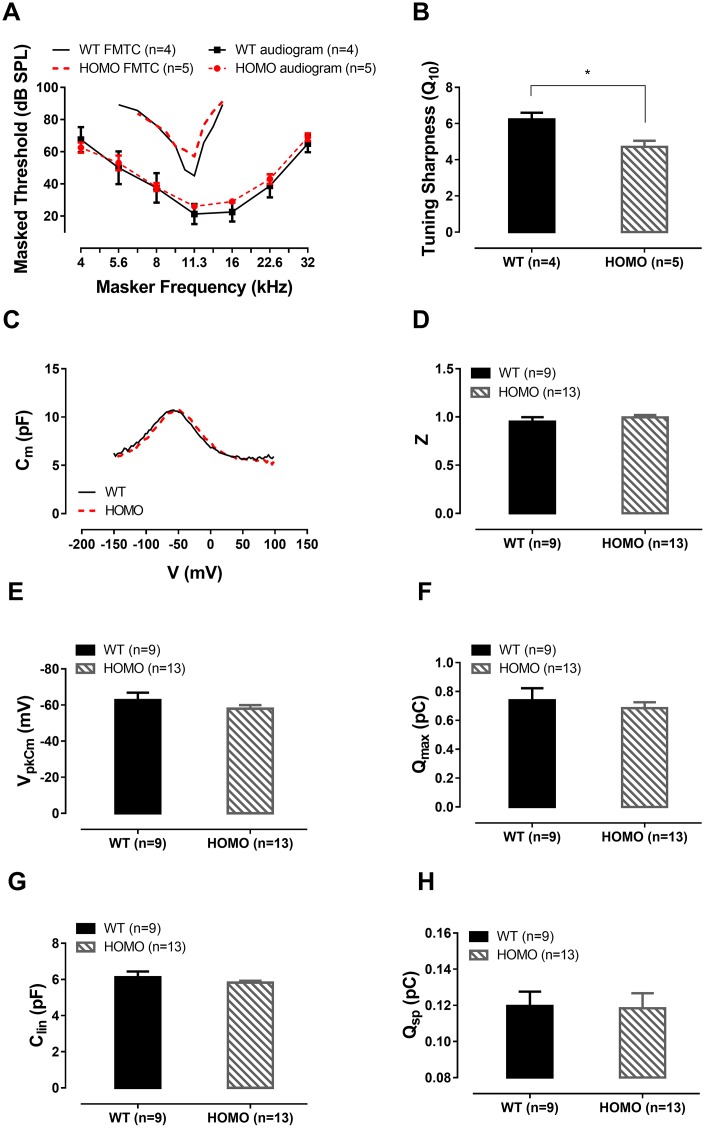
**ABR forward masking tuning curves and outer hair cell (OHC) patch clamp recordings in KI mice at 20 weeks.** (**A**) Homozygous mice exhibited no significant ABR threshold elevation (P>0.05, two-way ANOVA followed by Bonferroni post-test). Averaged ABR forward masking tuning curves of 11 kHz probe tone were also presented as a function of masker frequency. The averaged tip threshold of homozygous mice showed no significant difference compared to those of wild-type mice (P>0.05, Student’s unpaired t-test with Welch’s correction). The tail portions of the FMTC overlapped between the two genotype groups. Averaged audiograms from the same tested animals were provided for reference. (**B**) Significantly lower Q_10_ values measured in homozygous mice compared with their wild-type counterparts (6.228 ± 0.3705 and 4.712 ± 0.3353, Mean ± SEM for wild-type and homozygotes, respectively, *P=0.0204, Student’s unpaired t-test with Welch’s correction). (**C**) Representative OHC NLC traces from 20 week-old wild-type and homozygous mice. (**D**–**G**) No significant difference was found between the Z value, V_pkCm_, Q_max_ and C_lin_ of both genotypes (all P>0.05, Student’s unpaired t-test with Welch’s correction). (**H**) Normalized Prestin’s charge density Q_sp_, derived from Q_max_/C_lin_, showing no difference between the two groups (P>0.05, Student’s unpaired t-test with Welch’s correction).

We then evaluated the function of the cochlear amplifier by acquiring forward masking tuning curves (FMTC), a non-invasive approach substituting single auditory nerve fiber recording [41–44] for estimating OHC function and cochlear amplifier’s integrity. We chose 11.3 kHz as the probe frequency since this was one of the most sensitive frequencies. When tested in the 20 weeks old animals, no significant tip threshold difference, in line with the ABR threshold measurement, was found between two genotypes (Figure 5A, P>0.05 for ABR thresholds at all frequencies, two-way ANOVA followed by Bonferroni post-test; P>0.05 for tip thresholds of FMTC, Student’s unpaired t-test with Welch’s correction). Q_10_, also known as the sharpness of tuning and an indicator of cochlear amplifier function, was significantly reduced in the homozygotes (Figure 5B, *P= 0.0204, Student’s unpaired t-test with Welch’s correction), suggesting that the cochlear amplifier was indeed affected [45]. The tails of the tuning curves were virtually overlapping, indicating normal passive mechanics in homozygous cochleae.

Since the OHCs are the generator of the cochlear amplifier [46–50], we questioned whether the change in tuning was a result of reduced transduction driving force or altered prestin’s function that reduces the power of electromotility. We recorded in whole-cell patch clamped OHCs for nonlinear capacitance (NLC), a surrogate measurement of electromotility [51–53]. Results of NLC measurements detected no differences (Figure 5C–5H, all P>0.05, Student’s unpaired t-test with Welch’s correction). Taken together, these data did not support deteriorated OHC function being responsible for the observed phenotype. Changes in cochlear amplification could alternatively arise from either the reduced input from the transduction channel (lowered EP) or reactive shift of OHC operating point (voltage sensitivity of NLC and electromotility) [54] that failed to compensate.

### Altered IHCs function in KI mice

**Figure 6:**
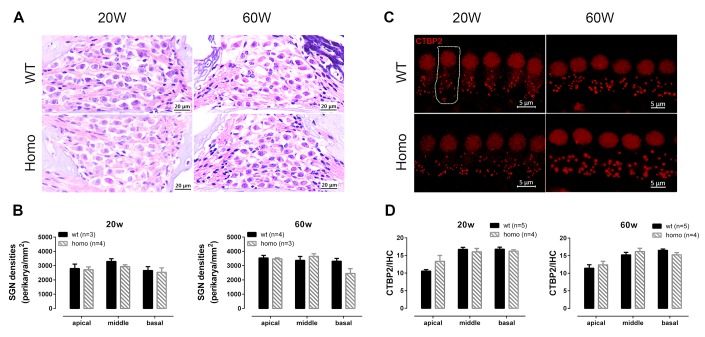
**Spiral ganglion neuron (SGN) and inner hair cell (IHC) synapse count in KI mice at different ages.** (**A**) Representative H&E staining images of SGNs at 20 and 60 week-old homozygous and wild-type mice (Scale Bar, 20 μm). (**B**) SGN density (Number of SGNs/area of Rosenthal’s canal) showing no significant difference between wild-type and homozygous mice at both time points (P>0.05 at both time points, two-way ANOVA followed by Bonferroni post-test). (**C**) Representative confocal images of IHC synapses. Dotted line outlines a single IHC (Scale Bar, 5 μm). (**D**) The numbers of CTBP2 puncta per IHC at both 20 and 60 weeks old showing no significant difference between two genotype groups (P>0.05 at both age groups, two-way ANOVA followed by Bonferroni post-test).

**Figure 7:**
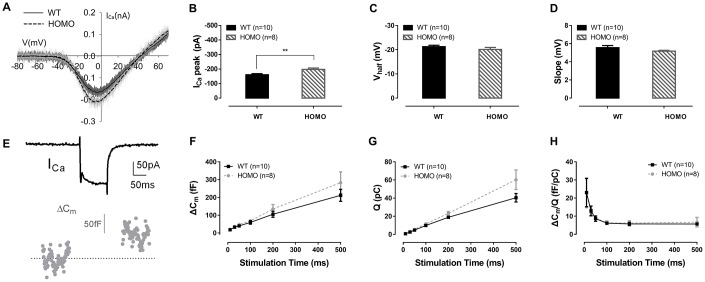
**Inner hair cell (IHC) patch clamp recordings in KI mice.** (**A**) Representative calcium currents (I_Ca_) induced by voltage ramps in IHCs of wild-type and homozygous mice. (**B**) The I_Ca_ peak of homozygous mice was significantly lower than wild-type mice (**P= 0.0091, t=2.978, df=15.49, Student’s unpaired t-test with Welch’s correction). (**C**) V_half_, and (**D**) slope of I_ca_ showed no significant difference between the two genotype groups (P>0.05, Student’s unpaired t-test with Welch’s correction). (**E**) Representative whole-cell I_Ca_ and membrane capacitance (C_m_) traces. Exocytosis was triggered by calcium current in response to a single step depolarization. (**F**) Homozygous mice exhibited no significant membrane capacitance change (ΔC_m_), (**G**) calcium charge (Q) or (**H**) ΔC_m_/Q, representing Ca^2+^ efficiency in triggering exocytosis (all P>0.05, two-way ANOVA followed by Bonferroni post-test).

Since prolonged latencies of ABR wave I were found in homozygous animals, we hypothesized that the synaptic transmission and nerve conduction between IHC and spiral ganglion neuron (SGN) might have been altered. H&E stainings showed no significant changes in SGN density between homozygous and wild-type mice at both 20 and 60 weeks of age (Figure 6A, 6B, all P>0.05, two-way ANOVA followed by Bonferroni post-test), excluding SGN as the underlying source of pathology. For the IHCs, we used confocal imaging for CTBP2 staining to assess changes of ribbon synapses. Quantified puncta of CTBP2 showed no difference between homozygous and wild-type mice at both 20 and 60 weeks (Figure 6C, 6D, all P>0.05, two-way ANOVA followed by Bonferroni post-test). Although there was no quantitative change in IHC ribbon synapses, the function of IHC could still have changed. To further evaluate IHC functions, we used whole-cell patch-clamp recording to measure their calcium current and exocytosis. We first applied voltage ramps to IHCs and recorded the calcium current. Results showed significantly larger calcium current (Figure 7A, 7B, P=0.0091, t=2.978, df=15.49, Student’s unpaired t-test with Welch’s correction) with normal V_half_ and slope (Figure 7C, 7D, both P>0.05, Student’s unpaired t-test with Welch’s correction) in homozygous IHCs. We then applied voltage steps to induce exocytosis and measured whole-cell capacitance before and after stimulation. We found that neither the capacitance change (ΔC_m_) nor the ratio of ΔC_m_ and the Ca^2+^ charge (ΔC_m_/Q) showed significant difference between the two genotypes (Figure 7F–7H, all P>0.05, two-way ANOVA followed by Bonferroni post-test), indicating that per vesicle release was unaffected.

### Environmental insults exacerbated cochlear pathology in KI mice, presenting them as risk factors for p.V37I patients

### p.V37I knock-in mice were more susceptible to noise exposure

In clinics, patients with p.V37I mutation exhibit varied onset and severity of hearing phenotypes, suggesting that environmental factors may accelerate the disease progression. For hearing, the primary source of environmental insult is noise [55]. When overstimulated, oxidative stress occurs, accompanied by excessive potassium release from hair cells into nearby perilymph [56–60]. With reduced ion permeability to allow potassium clearance and recycling, the cochlear function may be compromised. To test the susceptibility of p.V37I mutants to noise, we chose a 2 hour, 100 dB SPL 8-16 kHz bandpass noise to stress both homozygous and wild-type mice in part of the cochlea. Animals were tested at 20 weeks old (from 18 to 22 weeks), a time point prior to or at the onset of the hearing phenotype. The noise exposure we used was relatively mild, which only led to temporary threshold shift in controls [61]. This noise exposure paradigm would allow us to test whether homozygous animals are more susceptible to such environmental risks.

**Figure 8:**
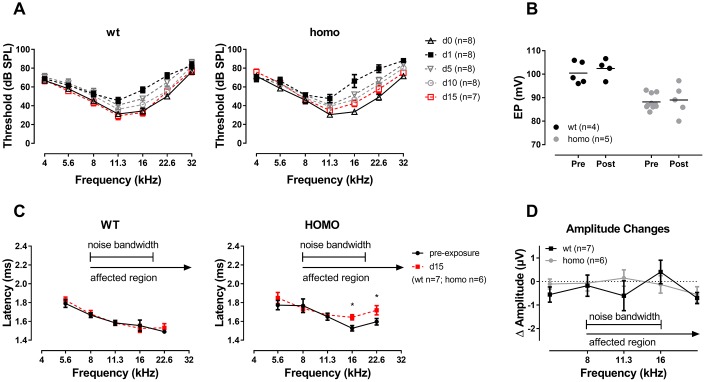
**ABR analysis and EP measurement in KI mice after noise exposure.** (**A**) ABR audiograms tracking changes of thresholds after one episode of a two-hour, 100 dB SPL, 8-16 kHz band-pass noise exposure. ABR thresholds of both groups recovered to the baseline level at 15 days after noise exposure (P>0.05 for both genotypes, two-way ANOVA followed by Bonferroni post-test). (**B**) EPs didn’t change in both wild-type and homozygous mice at day 15 of noise exposure (P>0.05, Student’s unpaired t-test with Welch’s correction). Baseline EPs were obtained by pooling data from young individuals less than 20 weeks old in Figure 3. (**C**) Comparison of Wave I latencies of day 0 (pre-exposure) and day 15 at 90dB SPL showing that homozygous mice presented a tendency of prolonged latency in frequencies above 11.3 kHz (*P=0.021, F_(1,5)_=11.003 at 16 kHz and *P=0.016, F_(1,5)_=12.961 at 22.6 kHz, two-way ANOVA followed by Bonferroni post-test). (**D**) Summary of ABR Wave I amplitude showing no significant difference between the two genotypes (P>0.05 for all frequencies, two-way ANOVA followed by Bonferroni post-test). ΔAmplitude=day 15 amplitude at 90 dB SPL - day 0 amplitude at 90 dB SPL.

**Figure 9:**
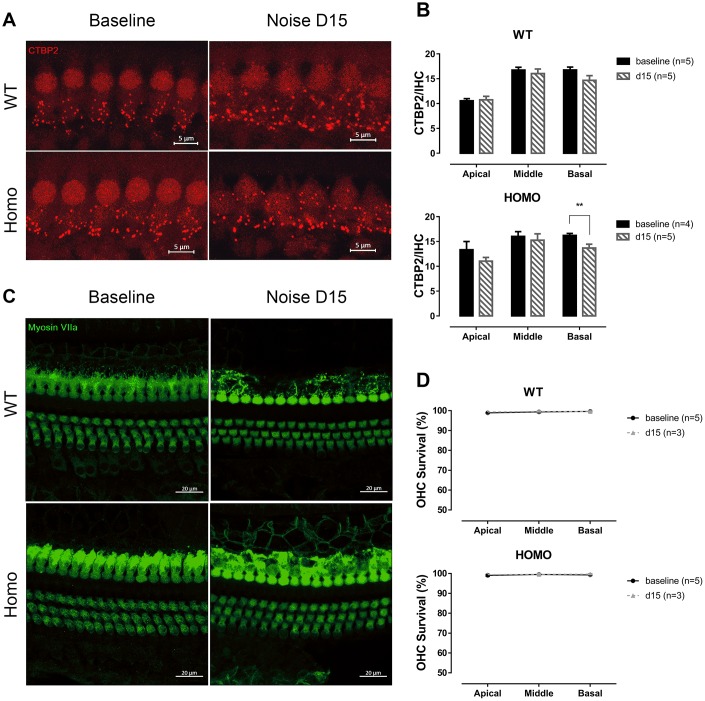
**IHC synapse and OHC count in KI mice after noise exposure.** (**A**) Representative confocal images of IHC CTBP2 puncta before and after noise exposure. (Scale Bar, 5μm). (**B**) Histograms summarizing averaged number of CTBP2 puncta at different turns (Mean + SEM). Reduction of CTBP2 was only observed at the basal turn in homozygous mice 15 days after noise exposure (*P=0.024, F_(1,4)_=12.423, two-way ANOVA followed by Bonferroni post-test). (**C**) Representative confocal images of hair cells before and after noise exposure. (Scale Bar, 20μm). (**D**) OHC survivals at day 0 and 15 were compared. No significant hair cell loss was observed in both genotypes in all three turns (all P>0.05, two-way ANOVA followed by Bonferroni post-test). Baseline images and data were obtained from the same group of 20-week-old animals as in Figure 6.

One day after the noise exposure, thresholds of the affected frequencies (above 11.3 kHz) elevated in both homozygous and wild-type mice, but to a higher degree in the homozygous mice. At 15 days after noise exposure, the averaged ABR threshold of homozygous mice were slightly higher than the baseline level but with no significance (especially at 16 kHz with 14.17dB difference and P=0.070), while wild-type mice completely recovered from the temporary threshold shift (Figure 8A, P>0.05 for all frequencies of both genotypes, two-way ANOVA followed by Bonferroni post-test). EP reduction was not essential to the observed susceptibility to noise since the EPs of both genotypes remained stable 15 days after noise exposure (Figure 8B, both P>0.05, Student’s unpaired t-test with Welch’s correction). ABR Wave I analysis at day 15 presented frequency dependent prolonged latencies (Figure 8C, P=0.021, F_(1,5)_=11.003 at 16 kHz and P=0.016, F_(1,5)_=12.961 at 22.6 kHz, two-way ANOVA followed by Bonferroni post-test) and normal amplitudes (Figure 8D, P>0.05 for both genotypes, two-way ANOVA followed by Bonferroni post-test) in homozygous mice compared to wild-type controls. CTBP2 puncta, a marker for ribbon synapse in HCs, were significantly reduced at the basal turns in homozygous mice at day 15 (Figure 9A, 9B, P=0.024, F_(1,4)_=12.423, two-way ANOVA followed by Bonferroni post-test). However, no hair cell loss was observed (Figure 9C, 9D, P>0.05 for both genotypes, two-way ANOVA followed by Bonferroni post-test). These changes in homozygous mice indicate that mutation in *Gjb2* rendered cochlea more vulnerable to noise exposure.

### Potassium accumulation in perilymph may be the underlying mechanism of the hearing loss of KI mice

**Figure 10:**
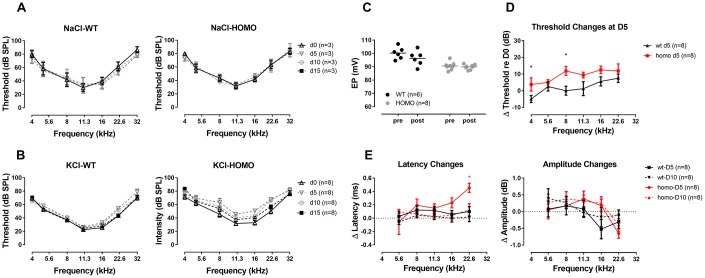
**ABR analysis and EP measurement in KI mice with a one-time trans-tympanic middle ear injection of KCl solution.** (**A**) ABR thresholds remaining unchanged throughout two weeks after the control injection of NaCl solution (all P>0.05 for both genotypes, two-way ANOVA followed by Bonferroni post-test). (**B**) More substantial ABR threshold shifts were observed in homozygous mice following a one-time, 150 mM KCl injection, and recovered at day 15 (P>0.05, two-way ANOVA followed by Bonferroni post-test). (**C**) EPs tested after direct round window membrane application of KCl solution for 10 minutes showing no significant change in both wild-type and homozygous mice (P>0.05, Student’s unpaired t-test with Welch’s correction). Baseline EPs were obtained by pooling data from young individuals less than 20 weeks old in Figure 3. (**D**) ABR threshold changes at day 5 after KCl injection (ΔThreshold=day 5 Threshold–day 0 Threshold) with a trend of larger threshold shift in homozygous mice (*P=0.047, F_(1,7)_=5.814 and *P=0.012, F_(1,7)_=11.332, for 4 kHz and 8 kHz, respectively, two-way ANOVA followed by Bonferroni post-test). (**E**) Summary of ABR Wave I latency changes (left panel, ΔLatency=averaged day 5 or day 10 Latency - averaged day 0 Latency) and Wave I amplitude changes (right panel, ΔAmplitude=day 5 or day 10 Amplitude at 90dB SPL - day 0 Amplitude at 90dB SPL). A frequency dependent increase in ΔLatency was observed in homozygous mice, especially in the frequency of 22.6 kHz at day 5 (*P=0.011, F_(1,7)_=11.727, two-way ANOVA followed by Bonferroni post-test). At day 10, latencies of wild-type mice almost recovered to the baseline level, while those of homozygous mice still had a residual latency prolongation at higher frequencies (P>0.05, two-way ANOVA followed by Bonferroni post-test). ΔAmplitude did not show any significant difference in all frequencies between two genotypes both at day 5 and day 10 (right panel, P>0.05, two-way ANOVA followed by Bonferroni post-test). Wave I responses for 32 kHz were missing therefore not included in this analysis.

**Figure 11:**
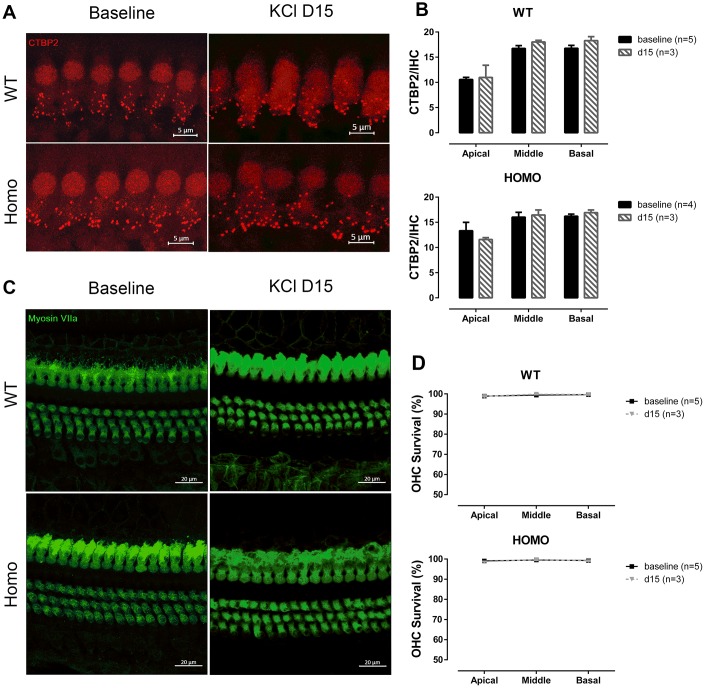
**IHC synapse and OHC count in KI mice after middle ear injection of KCl solution.** (**A**) Representative confocal images of IHC CTBP2 puncta before and after KCl middle ear injection. (Scale Bar, 5μm). (**B**) Histograms summarizing the average number of CTBP2 puncta at different turns (Mean + SEM). Middle ear injection of KCl solution did not affect CTBP2 expression (P>0.05, two-way ANOVA followed by Bonferroni post-test). (**C**) Representative confocal images of hair cells before and after KCl middle ear injection. (Scale Bar, 20μm). (**D**) Lack of significant hair cell loss observed in either genotype when compared to baseline data (d0) (P>0.05, two-way ANOVA followed by Bonferroni post-test). Baseline images and data were obtained from the same group of 20-week-old animals as in Figures 6 and 9.

The excitatory toxicity to IHCs after noise exposure offered us a clue that cochlear pathology may arise from excessive potassium passage and extracellular accumulation, either from over-stimulating hair cells or from ineffective clearance due to attenuated GJ function [23, 62]. To test if homozygous mice have lower potassium clearance capability, we performed a one-time trans-tympanic injection of 150 mM KCl solution (150mM NaCl as control) into the middle ear cavity in 20 weeks old (from 18 to 22 weeks) animals. Neither wild-type nor homozygous mice responded to NaCl injection (Figure 10A, P>0.05 for both genotypes, two-way ANOVA followed by Bonferroni post-test), ruling out the impact of the procedure and Cl^-^. When given 150 mM KCl, the potassium ion permeated through the round window membrane served as K^+^ shock that stressed the recycling process. Post-injection ABR thresholds elevated in homozygous mice and almost recovered after 15 days (Figure 10B, P>0.05, two-way ANOVA followed by Bonferroni post-test). These changes were not EP derived because direct application of 150 mM KCl solution onto the round window did not reduce EP (Figure 10C, P>0.05, Student’s unpaired t-test with Welch’s correction). By comparing the ABR threshold changes at day 5, we found that homozygous mice had significantly larger shifts (Figure 10D, P=0.047, F_(1,7)_=5.814 and P=0.012, F_(1,7)_=11.332, for 4 kHz and 8 kHz, respectively, two-way ANOVA followed by Bonferroni post-test). Wave I analysis of homozygous mice also revealed prolonged latencies in high-frequency regions at day 5 (Figure 10E, left panel, P=0.011 and F_(1,7)_=11.727 at 22.6 kHz, two-way ANOVA followed by Bonferroni post-test), while amplitudes were unaffected (Figure 10E, right panel, P>0.05, two-way ANOVA followed by Bonferroni post-test). Although KCl injection could affect the Wave I latencies of homozygous mice, the number of synapses did not change at 15 days post-injection (Figure 11A, 11B, P>0.05, two-way ANOVA followed by Bonferroni post-test). No significant OHC loss was observed both in non-treated mice (baseline) and at 15 days after KCl injection (Figure 11C, 11D, P>0.05 between the two time points, two-way ANOVA followed by Bonferroni post-test). Taken together, we conclude that excessive potassium accumulation in perilymph was a risk factor in the development of pathology in *Gjb2* mutation.

### KI mice were sensitive to furosemide insult, an NKCC inhibitor

**Figure 12:**
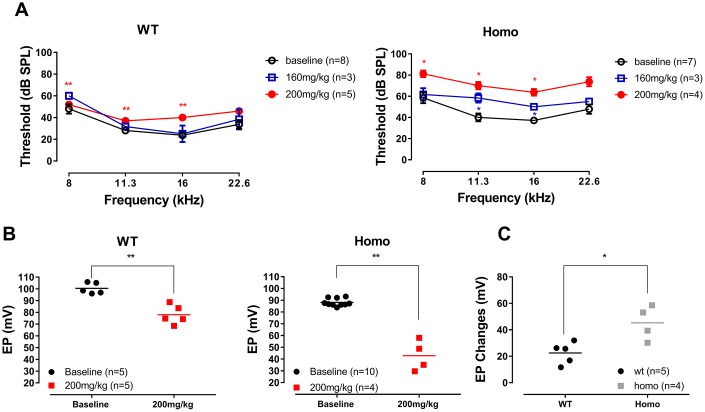
**ABR and EP measurement before and after furosemide treatment.** (**A**) ABR thresholds of four representative frequencies (8, 11.3, 16 and 22.6 kHz) monitored 20 to 30 minutes after furosemide injection. No threshold shift was observed in wild-type mice when 160 mg/kg furosemide was administered (all P>0.05, two-way ANOVA followed by Bonferroni post-test), and only a mild threshold elevation occurred when the dose was increased to 200 mg/kg (left panel, **all P<0.01 at 8, 11.3 and 16 kHz, two-way ANOVA followed by Bonferroni post-test). Homozygous mice responded to 160 mg/kg furosemide with moderate threshold elevation (right panel, *P=0.038, F_(1,2)_=25.00 at 11.3 kHz and P=0.020, F_(1,2)_=49.00 at 16 kHz, two-way ANOVA followed by Bonferroni post-test), and hearing loss developed when the dose increased to 200 mg/kg (right panel, *all P<0.05 at 8, 11.3, 16 kHz, two-way ANOVA followed by Bonferroni post-test). (**B**) EP measurements in the same animals 2 hours after the 200mg/kg furosemide injection (**P=0.0014, t=5.376 and df=6.403 for wild-type and P=0.0053, t=6.932 and df=3.149 for homozygous mice, Student’s unpaired t-test with Welch’s correction). Baseline EPs were collected from the age-matched mice from Figure 3. (**C**) EP changes in both groups compared to the averaged baselines of non-treated controls younger than 20 weeks old. Homozygous mice had a significantly larger EP reduction than wild-type mice (*P= 0.0287, t=3.084, df=4.825, Student’s unpaired t-test with Welch’s correction).

The ~12 mV average EP reduction measured in homozygous may not be sufficient to drop the hearing threshold. However, the slightly reduced EP indicates that the generator of EP may operate at a marginal condition that is vulnerable to the insults of ototoxic drugs. Diuretic drug furosemide causes hearing loss by suppressing NKCC (Na-K-Cl cotransporter) in stria basal cell [63–66]. We therefore reasoned that furosemide could be a potential risk for homozygous mice as its impact may be exacerbated in an already vulnerable *Gjb2* mutation carrier. Here we used two different doses of furosemide, 160 mg/kg and 200 mg/kg intraperitoneal injection, to stress mice of both genotypes (from 18 to 22 weeks) and then monitored the ABR threshold elevations at 4 representative frequencies: 8, 11.3, 16 and 22.6 kHz. At 30 minutes after injection, wild-type mice resisted to the dose of 160mg/kg, while the ABR threshold of homozygous mice began to rise at 11.3k and 16 kHz (Figure 12A, blue line, all P>0.05 for wild-type; while for homozygous mice, P=0.038, F_(1,2)_=25.00 at 11.3 kHz and P=0.020, F_(1,2)_=49.00 at 16 kHz, two-way ANOVA followed by Bonferroni post-test). When the dose increased to 200mg/kg, a mild but significant threshold elevation was observed in wild-type animals whereas homozygous mice showed profound hearing loss with threshold elevations exceeding 30 dB SPL (Figure 12A, red line, all P<0.01 at 8, 11.3 and 16 kHz for wild-type mice and all P<0.05 at 8, 11.3, 16 kHz for homozygous mice, two-way ANOVA followed by Bonferroni post-test), indicating that homozygous mice had a lower tolerance to furosemide insult. To further validate this observation, at 2 hours after furosemide injection, EPs were measured. Although both genotypes showed significant EP reduction compared to their uninjected controls (from 100.48 mV to 78.00 mV in wild-type mice, and from 88.19 mV to 42.88 mV in homozygous mice) (Figure 12B, P=0.0014, t=5.376 and df=6.403 for wild-type; P=0.0053, t=6.932 and df=3.149 for homozygous mice, Student’s unpaired t-test with Welch’s correction), the homozygous mice exhibited more substantial EP drop (EPs drop 22.48 mV vs. 45.32 mV in wild-type and homozygous mice, respectively) (Figure 12C, P= 0.0287, t=3.084, df=4.825, Student’s unpaired t-test with Welch’s correction).

## DISCUSSION

### Consideration of the animal model

We have uncovered the underlying cochlear pathology of this mild form of *Gjb2* mutation. Although the identified pathologies were in line with the predicted changes of similar *Gjb2* mutations [8, 25, 67, 68], the severity of disease progression was not in congruence with human disease. This is in part due to the controlled, mostly quiet acoustic environment where the diseased animals were raised; with lack of exposure to environmental risks, disease progression has taken a protracted timeline in our KI mouse model. With the introduction of environmental insults that were specially designed to stress the potassium recycling pathway, the progression of hearing phenotype in KI mice was accelerated. Below we discuss the tissue-specific cochlear pathology and the risk factors identified to have accelerated the progression of hearing losses.

### Cochlear pathology

### Consideration of EP

The primary function of sensory hair cells is to transduce the mechanical movement of the basilar membrane into electrical changes. Both IHCs and OHCs are involved with this transduction process initiated by the deflection of stereocilia: IHCs serve as a true sensor of sound stimuli while OHCs act as a mechanical amplifier [69, 70]. The transduction process is achieved through the inflow of K^+^ into stereocilia powered by the positively charged EP. SV is the generator of the EP and is involved with the energy consuming Na-K-ATPase, NKCC and a number of ion channels including Kir4.1 [71–75]. Atrophy in SV and lateral wall fibrocytes is associated with declined EP in gerbils [76]. GJs such as Cx26 and Cx30 electrically couple the fibrocytes from the spiral ligaments with the basal cells of SV [30], forming functional unity to deliver potassium ion into intrastrial space then scala media to produce EP [77–81]. K^+^ entered hair cells through transduction channels are subsequently removed from hair cells by a number of potassium channels at the base of hair cells, then re-absorbed by the mechanically coupled supporting cells [62, 80].

GJs are mostly distributed in the cochlear supporting cells to form intercellular channels that electrically couple the supporting cells, allowing the exchange of ions and other small molecules among the coupled cells [10, 11]. The primary function of GJ is believed to form pathways for potassium recycling in the cochleae [62, 77]. Potassium recycling dysfunction has been considered as the primary pathogenesis of GJ related hearing loss [35]. *In vitro* measurement of p.V37I, the mutation that forms intercellular channels showed reduced function [82], indicating that the mutant cochleae may operate with reduced potassium clearance capability.

In our measurements, lower EP was consistent throughout the mutant animal’s lifespan, indicating a compromised EP generation process. Lower permeability of GJs decreased potassium transported to the SV, which, in combination with NKCC inhibition from furosemide [63, 83, 84], significantly dropped EP in homozygous mice. In our case, the mutant Cx26 channels were more vulnerable to furosemide insult, responding with elevation in ABR threshold and corresponding EP reduction at a relatively low dose.

### What drops the cochlear amplifier?

At up to 3-4 months of age, homozygotes were able to maintain normal cochlear function as seen by standard ABR threshold measurements. The primary contributor to the sensitive hearing threshold is the cochlear amplifier [49, 85]. OHC electromotility, the power source of the cochlear amplifier [48, 86–89], is subjected to efferent regulation [90, 91]. Two types of efferent innervations control the function of OHC: GABAergic and Cholinergic efferent fibers that modulate intracellular Cl^-^ concentration and potassium current of the OHCs, respectively [92, 93]. Assuming the impact of potassium accumulation under the base of OHC can be offset by increased small conductance (SK) potassium current from activation of Cholinergic fibers [94–98], OHC electromotility could still be maintained at the optimal resting potential. However, maintenance of resting membrane potential may come at the expense of causing more potassium out-flow to extracellular space that further stresses the potassium recycling.

Our FMTC measurements showed that at the onset of hearing threshold elevation, the cochlear amplifier was indeed compromised. The reduced Q_10_ indicates that OHCs were either operating with reduced transduction driving forces or compromised prestin function, i.e., the voltage sensitivity shifted so that the generator of the cochlear amplifier could not function at the best level to boost basilar membrane vibration, an extreme example of which is found in prestin 499 KI mice [69, 99, 100]. There are many biophysical factors that could shift voltage sensitivity such as resting membrane potential (also termed as ‘prepulse effect’) [101, 102], intracellular chloride concentration, membrane tension and temperature [102]. Two potential scenarios are that 1) extracellular potassium accumulation at the base of OHCs shifts resting membrane potential in depolarizing direction and 2) lower EP reduces transduction driving force. Both scenarios would require hyperpolarizing efferent regulation to compensate. This compensatory mechanism worked initially up to the first 3-4 months of the animal’s life. However this state of ‘normality’ was fragile. Loss of compensatory power eventually failed to maintain optimal cochlear amplification in KI mice.

In individual isolated OHCs at the age of 5 months, NLCs of homozygotes OHCs were identical to those of wild-type controls. This means at this time of disease progression, individual OHC function was not compromised, thereby ruling out the molecular changes of prestin as the cause of reduced cochlear amplifier.

### Inner hair cell pathology

IHCs depend on transduction progress to activate synaptic transmission. The transduction current from stereocilia depolarizes the IHC, leading to the activation of calcium current that triggers the synaptic release [103–105]. Meanwhile, potassium channels are activated in the basal portion of the IHC to rebalance the intracellular ion composition. Unlike OHCs, IHCs lack the direct efferent innervation [106]. When mutant GJs operate on the reduced level that fails to recycle potassium promptly, K^+^ accumulation in perilymph around hair cells may occur, leading to depolarization and excitotoxic damage manifested as ABR Wave I latency changes and eventually hearing loss.

According to our data, short-term potassium accumulation from KCl middle ear injection only caused temporary damage to the IHCs. But, when other perturbation mechanisms like oxidative stress were involved or the duration of exposure extended, the damage to the hearing may become permanent. Patch clamp recordings of IHCs showed increased calcium currents and △Cm, suggesting that the changes of IHC function were of perilymph origin.

### Progression of hearing loss under environmental insults - clues for disease prognosis and prevention

Unlike the mouse model that grows in a controlled, quiet environment, the hearing of patients with homozygous p.V37I mutation varies significantly, including different onset timepoints and severity of hearing loss. [17, 107]. Our results imply that environmental factors, like noise, may be responsible for the phenotype variation in human *GJB2* mutations. People of different occupations and lifestyles are exposed to varying levels of stresses, leading to accumulative damage to hearing and, when unable to compensate, accelerating the progression of hearing losses. Other risk factors, such as ototoxic drugs, oxidative stress, and modifier genes, may also be involved in varying disease progression [29, 66, 108, 109]. Because the *GJB2* mutation related hearing loss is late-onset, there remains a time window for both prevention and therapeutic intervention. In theory, removing/mitigating the environmental stress or repairing the function of GJ during this time window could delay the disease onset or reverse the hearing phenotype with appropriate countering methods. Further studies are needed to better understand the GJ related hearing losses and to develop potential therapeutic intervention options. Below we discuss these risk factors explored in more detail:

1) Noise, as the primary source of risk to hearing, can be identified and prevented by avoidance. However, prevention is not as easy as offering a general guideline, especially when speech and music are essential in our daily life. It is, therefore, crucial to assess the dose of noise that crosses the threshold of being harmful to the vulnerable subjects. The impact of noise exposure identified in our study is comparable to one episode of the concert-going experience. The eventual permanent damage is coined as ‘hidden hearing loss’ [110, 111]. So far this phenomenon has been mainly observed in murine species. It remains controversial whether the hidden hearing loss occurs in human [112]. Nonetheless, a careful audiological and epidemiological assessment in the human will certainly allow better guidance to be formulated for patients.

2) Hair cells in our study differentially responded to potassium surge from middle ear injection of KCl application: OHCs from both genotypes maintained their normal functions as seen in their near-total recovery of ABR thresholds while IHCs did not recover as shown in frequency-dependent latency prolongation. Because the initial ABR threshold elevations were across all frequencies in both genotype groups, we can rule out the possibility of diffusion derived changes, i.e., ions permeated through round windows hit first at the high-frequency base region. The differential responses between the two types of hair cells may result from their different efferent innervation patterns. The change of IHC function manifested by the calcium current change and prolonged ABR wave I latency that may arise from extracellular potassium accumulation, suggesting that the temporal coding of sound may be compromised. It is conceivable that speech perception in *GJB2* mutation patients may change due to the IHC pathology which begs further investigations.

### Clinical perspectives

Gene therapy has recently gained successes in treating various kinds of deafness [33, 113–118]. With the advances in gene therapy delivery and safety such as base editing [27, 119, 120] and improved encapsulation via nanoparticles or hydrogels [121–123], we are optimistic that treatment options for patients will become more available in the not too distant future. Meanwhile, chemicals that target the potassium channel activity also remain a promising therapeutic approach to reduce or delay the disease progression. With some successes in middle and inner ear applications [124, 125], trans-tympanic injection with hydrogel encapsulation method may become a first line noninvasive therapy that allows either genetic materials or therapeutic chemicals to reach the inner ear space. Finally, identifying environmental risk factors that compromise the cochlear potassium recycling would allow prevention of disease progression by avoidance, a future direction for us to pursue.

## MATERIALS AND METHODS

### Animals

Homozygous p.V37I knock-in mice (homozygous mice) and their age-and-sex-matched 129T2/SvEmsJ wild-type control mice (wild-type mice) were used in this study. The generation (using embryonic stem cell gene targeting) and genotyping of this mouse model were described in our previous publication [26]. The genotype was confirmed by sequencing of the mouse tail genomic DNA. All mice were originally bred from two heterozygous breeders. Then, wild-type non-carriers and homozygous mice were bred separately.

### Auditory Brainstem Response (ABR)

ABRs were recorded from mice anesthetized with chloral hydrate (480 mg/kg, IP) (Sigma Aldrich-Fluka, St. Louis, MO, USA). Body temperature was maintained at 37°C throughout recording with a Homeothermic Monitoring System (Harvard Apparatus, 55-7020). Three needle electrodes were positioned sub-dermally at the vertex (active), right mastoid region (reference), and the left shoulder (ground). A short toneburst of 3 ms duration with 1 ms rise and fall time was generated by the RZ6 workstation (Tucker–Davis Technologies, Alachua, FL, USA). Stimulus sounds were presented free-field via an MF1 speaker (TDT) placed 10cm away from the vertex. Stimulus frequencies of 32 kHz to 4 kHz were presented in half-octave step. The sound level was decreased from 90 to 0 dB SPL in 5-dB steps. Stimulus presentation rate is 20 per second. 400 responses were averaged at each frequency each level. Thresholds were determined by minimal stimulus level that evoked any one of the initial four peaks. Near threshold recordings were repeated to confirm the findings.

All latencies and amplitudes of ABR peak I were measured and analyzed by using BioSigRZ software (TDT). Latency referred to the time from the onset of the stimulus signal to the peak, while amplitude was determined by averaging the △V of both sides of the peak.

### Forward masking tuning curve

Mice, speakers, and electrodes were prepared the same as those in the ABR test. Because of extended recording time is required, supplement doses of Chloral Hydrate (240 mg/Kg, IP) were administered when needed. Stimulus signals were generated by using SigGenRZ (TDT). The stimulus consists of a 50 ms masker tone (1 ms rise and fall, Channel A), followed by a 10 ms quiet period, then a 3 ms probe tone presented at 60ms after the onset of the masker (Channel B). The signals from two channels were integrated by SM5 Signal Mixer (TDT) then deliver to an MF1 speaker. Presentation rate was set at 8 times per second to avoid fatigue. Probe tone level was set to evoke ~1 μV wave I peak amplitude (usually around 15 dB above threshold). Masker level started from 90 dB SPL and descended in 5-dB step. The threshold is determined as the level of which 50% wave I amplitude reduction from the probe alone paradigm was achieved. Q_10_ value, reflecting the sharpness of tuning, was computed by dividing probe frequency by the bandwidth of tuning curve measured at 10 dB above tip threshold [41].

### Endocochlear potential

Mice were anesthetized with ketamine (150 mg/kg, IP) and xylazine (6 mg/kg, IP). Body temperature was maintained at 37°C on a heating operating table (Harvard Apparatus, 73-3771). A mouse head holding adaptor (MA-6N, Narishige, Tokyo, Japan) was used to maintain a supine position. A tracheotomy was performed, followed by opening the auditory bulla through a ventral approach to expose the round window of the cochlea. A silver ground electrode coated with silver chloride was placed under the skin. A microelectrode (9 to 16 MΩ; 1B150F-4; World Precision Instruments, Sarasota, FL, USA) filled with 3 M KCl was mounted on a motorized manipulator (IVM Single, Scientifica Limited, East Sussex, UK). An Axopatch 200B amplifier (Molecular Devices, LLC., San Jose, CA, USA) was used for current clamp recording with an Axon Digidata 1550B and interfaced by software pCLAMP (version 10.6, Molecular Devices, LLC., San Jose, CA, USA). The microelectrode was inserted through the round window membrane into the scala tympani and then advanced through the basilar membrane into the scala media to measure EP. To confirm the electrode is in scala media, we used following series of procedures: 1) withdrawing into the scala tympani, 2) re-entering scala media and continuing advancing into scala vestibuli and 3) retreating back to scala tympani. The voltage measured in scala tympani was adjusted to 0 mV as the baseline. The first stable value measured in scala media was EP. Mice were euthanized after completion of the measurement.

### Noise exposure

Mice were anesthetized with chloral hydrate (480 mg/kg, IP) (Sigma Aldrich-Fluka, St. Louis, MO, USA) to test the baseline ABR (pre-exposure) and then placed into a custom made wire cage on a heating pad (Harvard Apparatus, 55-7020) with the temperature maintained at 37°C. A free-field MF1 speaker positioned 10 cm away from the vertex of the mice presented an 8-16 kHz band-pass noise generated by the RZ6 workstation (Tucker–Davis Technologies, Alachua, FL, USA). Noise exposure was performed at 100 dB SPL for two hours. ABRs were repeated at before noise exposure (day 0) and at day 1, 5, 10 and 15 after noise exposure. ABR waves were analyzed on day 0 and day 10. Post-exposure latencies and amplitudes were compared between two genotype groups (subtracting levels of day 10 from day 0).

### Trans-tympanic middle ear injection

After anesthetized with chloral hydrate (480 mg/kg, IP) (Sigma Aldrich-Fluka, St. Louis, MO, USA), mice were placed in a lateral position under a surgical microscope (OPMI VARIO 700, Carl Zeiss Meditec AG, Jena, Germany). 150 mM NaCl or KCl solution (300 mOsm) was injected through the tympanic membrane into the middle ear (~5μl for each ear) using a 33 gauge needle with a calibrated 25 μl syringe (7803-05; CAL7654-01, Hamilton Company Inc., Reno, Nevada, USA). Mice were recovered on a heating pad in a supine position until restored free activities. ABRs were tested before injection (day 0) and at 5, 10 and 15 days after injection. ABR wave I was analyzed at day 0, 5 and 10 after KCl injection. Latencies and amplitudes changes (subtracting level of day 0 as the baseline from post-exposure days) were compared between the two genotypes.

### Furosemide treatment

Age-matched male homozygous and wild-type mice were selected for the furosemide treatment. After anesthetized with chloral hydrate (480 mg/kg, IP) (Sigma Aldrich-Fluka, St. Louis, MO, USA), baseline ABRs were measured, followed by an intraperitoneal injection of furosemide (Sigma Aldrich-Fluka, St. Louis, MO, USA) of two different doses (160 mg/kg or 200 mg/kg). ABR thresholds at frequencies 8, 11.3, 16 and 22.6 kHz were monitored until the threshold elevations were stable (around 20 to 30 minutes after furosemide injection). Around 2 hours after furosemide injection, EPs were measured.

### Tissue preparation

Animals were euthanized, and then cochleae of both sides were harvested, with each cochlea dedicated to one particular morphological assessment (one cochlea per animal per analysis). The Cochleae were quickly perfused with 4% PFA through the punctured round and oval windows. A small opening was then made at the apex and cochleae were left in 4% PFA overnight. After fixation for overnight at 4°C, cochleae were decalcified with 10% EDTA in phosphate buffered saline for 4 days at room temperature and then sensory epithelium was dissected and cut into three turns for further immunofluorescent staining.

For H&E staining, the decalcified cochleae were then taken through graded dehydration. Slices were cut through the modiolus with 4μm thickness. Slices containing 3-4 Corti’s organs were chosen for H&E staining.

### Immunofluorescent staining, confocal imaging, and H&E staining

For immunofluorescent staining, specimens were permeabilized and blocked with 0.25% Triton and 10% BSA mixture for 60 minutes at room temperature. After incubating with rabbit anti-Myosin VIIa (1:300, 25-6790, Proteus BioSciences Inc, Ramona, CA, USA) and mouse (IgG1) anti-CTBP2 (1:300, 612044, R&D Systems, Inc., Minneapolis, MN, USA) or rabbit anti-connexin 30 (71-2200, Thermo Fisher Scientific, Inc., Waltham, MA, USA) and mouse (IgG2a) anti- connexin 26 (33-5800, Connexin 26, Thermo Fisher Scientific, Inc., Waltham, MA, USA) at 4°C overnight, all tissues were rinsed 3 times in PBS followed by incubation with secondary antibody Alexa Fluor 488-conjugated goat anti-rabbit (1:300, R37116, Sigma-Aldrich, Inc., St. Louis, MO, USA), Alexa Fluor 647 goat anti-mouse (IgG1) (1:300, A21240, Molecular Probes, Waltham, Massachusetts, USA), and Alexa Fluor 555 goat anti-mouse (IgG2a) (1:1000, A21137, Molecular Probes, Waltham, Massachusetts, USA) at room temperature for 2 hours. After washed thrice in PBS, specimens were mounted in ProLong® Gold Antifade Reagent (P10144, Thermo Fisher Scientific, Inc., Waltham, MA, USA) on a glass slide. Images were acquired using a Zeiss LSM 880 laser confocal microscope (Carl Zeiss Microscopy, Jena, Germany). CTBP2 puncta per IHC, OHC loss, and length of GJs were measured in 3-4 different regions of every turn of the cochlea. CTBP2 count and 3D-reconstruction of GJs were accomplished using Imaris software (Bitplane AG, Zurich, Switzerland). Length of GJ was measured using ImageJ software (Wayne Rasband, National Institutes of Health, Bethesda, Maryland, USA), and 4 GJs (including the longest and shortest) were averaged in 4 regions of every turn [126]. All confocal images were pseudocolored.

Midmodiolar sections were stained with H&E, and images were analyzed using software ImageJ (Wayne Rasband, National Institutes of Health, Bethesda, Maryland, USA). The SGN density in Rosenthal’s canal of every turn was counted by calculating the number of SGNs per um^2^. Morphology of stria vascularis was evaluated by measuring the cross-sectional area of each turn.

### Whole-cell patch clamp recordings

Mice were euthanized by overdose injection of Chloral Hydrate. Temporal bones were then dissected, and cochlea bones were stripped in the extracellular solution for either IHC or OHC recording. All recordings were performed on dissected mouse cochlear explant at ~20% normalized distance from the apex, corresponding to frequencies of 8-16 kHz. An Axopatch 200B patch clamp amplifier (Molecular Devices, LLC., San Jose, CA, USA) and Digidata 1440B interface were used for the experiment. Micropipettes were pulled from borosilicate glass capillaries (1B150-4, World Precision Instruments, Inc., Sarasota, FL, USA) using a micropipette Puller (P2000, Sutter Instrument, Novato, CA, USA) with pipette resistances ranging from 4–6 MΩ. All recordings and analysis were performed using jClamp software (http://www.scisoftco.com/jclamp.html, New Haven, CT, USA).

For IHC capacitance and calcium current measurements, the intracellular solution contained the following (in mM): CsMSF 105, CsCl 20, HEPES 10, TEA-Cl 10, EGTA 2, Mg-ATP 3, and Na-GTP 0.5, pH 7.2~7.4 with CsOH, using D-Glucose to bring osmolality to 300 mOsm. The sensory epithelium was isolated and dissected in extracellular solution as follow (in mM): NaCl 105, KCl 2.8, TEA-Cl 35, CaCl_2_ 5, MgCl_2_ 1 and HEPES 10, pH 7.2~7.4 with NaOH, using D-glucose to adjust osmolality to 310 mOsm.

To examine calcium channel activation, we applied a voltage ramp from -80 mV to +70 mV (500 ms) to IHCs under voltage-clamp and recorded the resulting calcium current (I_Ca_). The current-voltage relationship was fitted with the following equation:

$$\text{I}(\text{V})=(\text{V}-{\text{V}}_{\text{rev}})\times \frac{{\text{G}}_{\text{max}}}{1+\text{exp}(-(\text{V}-{\text{V}}_{\text{half}})/k)}$$

where V is the membrane potential, V_rev_ is the reversal potential, G_max_ is the maximum conductance, V_half_ is the half-activation voltage, and the slope (k) indicates the steepness of voltage dependence. The I_Ca_ peak was also determined and compared between IHCs of different genotypes.

To examine exocytosis in IHCs, we turned to capacitance measurements. IHCs were holding at -80 mV with continuous high-resolution two-sine waves (390.6 and 781.2 Hz, 20 mV) superimposed to measure whole-cell capacitance before and after voltage steps that were applied to induce exocytosis. The averaged capacitances before and after the depolarizing pulses were subtracted for capacitance change: △C_m_ = C_m (response)_ - C_m (baseline)_. Series of depolarizing pulses with durations of 10ms, 20ms, 50ms, 100ms, 200ms, and 500ms were used. △C_m_ was measured and compared with Ca^2+^ current charge (Q) to evaluate vesicle release.

For OHCs nonlinear capacitance (NLC) measurements, the extracellular solution was as follow (in mM): NaCl 132, CaCl_2_ 2, MgCl_2_ 2, HEPES 10, pH 7.2~7.4 with NaOH, using D-Glucose to bring osmolality to 300 mOsm. The intracellular solution was the same as the extracellular solution except with the addition of 10 mM EGTA. Membrane holding potential was set at 0 mV. A 10 mV continuous high-resolution two-sine stimulus (390.6 and 781.2 Hz) superimposed onto a 300 ms voltage ramp (from +160 to -160 mV) was used [51]. The first derivative of a two-state Boltzmann function was used to fit all capacitance data [53],

$$\begin{array}{c} C_{m}=Q_{\max}\frac{ze}{kT}\frac{b}{{\left(1+b\right)}^{2}}+C_{lin}\;\;\;\;\;\text{where}\\ b=\exp\left(\frac{-ze\left(V_{m}-V_{pkCm}\right)}{kT}\right) \end{array}$$

Where Q_max_ is the maximum nonlinear charge moved, V_pkcm_ is the voltage at peak capacitance or equivalent to half maximum charge transfer, V_m_ is membrane potential, z is valence, C_lin_ is the linear membrane capacitance, e is electron charge, k is Boltzmann’s constant, and T is absolute temperature.

### Statistics

All averaged data are presented as mean±SEM from at least 3 independent measurements. One cochlear per mouse was used per test, i.e. EP and morphological assessments. Statistical analysis of the data was performed using Student’s unpaired t-test with Welch’s correction for comparisons between wild-type and homozygous mice (GraphPad Prism 7, GraphPad Software, Inc., San Diego, US) or two-way ANOVA followed by Bonferroni post-test for comparisons between genotypes that involves frequency, sound level and cochlear turn (SPSS 25, IBM Corp., New York, US). For all statistical analysis, results were considered significant when P<0.05.

### Study approval

The experimental protocol was approved by the Ethics Committee of Ninth People’s Hospital, Shanghai Jiao Tong University School of Medicine and performed in accordance with the guideline for experimental animal welfares of Ninth People’s Hospital, Shanghai Jiao Tong University School of Medicine.
